# The Role of Platelet Dysfunctions in the Pathogenesis of the Hemostatic-Coagulant System Imbalances

**DOI:** 10.3390/ijms26062756

**Published:** 2025-03-19

**Authors:** Oana-Viola Badulescu, Manuela Ciocoiu, Maria Cristina Vladeanu, Bogdan Huzum, Carmen Elena Plesoianu, Dan Iliescu-Halitchi, Andrei Bojan, Codruta Iliescu-Halitchi, Iris Bararu Bojan

**Affiliations:** 1 Department of Pathophysiology, Morpho-Functional Sciences (II), Faculty of Medicine, University of Medicine and Pharmacy Gr. T. Popa, 700115 Iasi, Romania; oana.badulescu@umfiasi.ro (O.-V.B.); manuela.ciocoiu@umfiasi.ro (M.C.); iris.bararu@umfiasi.ro (I.B.B.); 2 Department of Orthopedics and Traumatology, Surgical Science (II), Faculty of Medicine, University of Medicine and Pharmacy Gr. T. Popa, 700115 Iasi, Romania; 3 Department of Internal Medicine, Faculty of Medicine, University of Medicine and Pharmacy Gr. T. Popa, 700115 Iasi, Romania; carmen-elena.plesoianu@umfiasi.ro (C.E.P.);; 4 Department of Surgical Sciences, Faculty of Medicine, University of Medicine and Pharmacy Gr. T. Popa, 700115 Iasi, Romania; 5 Department of Pediatry, Faculty of Medicine, University of Medicine and Pharmacy Gr. T. Popa, 700115 Iasi, Romania; olimpiada.iliescu@umfiasi.ro

**Keywords:** platelet dysfunction, the hemostatic-coagulant system, hemostasis, thrombosis

## Abstract

Platelet dysfunction plays a critical role in the pathogenesis of various disorders affecting the hemostatic-coagulant system. This review aims to explore the mechanisms by which platelet dysfunctions contribute to the disruption of hemostasis, leading to an increased risk of both thrombosis and bleeding. Platelets, traditionally known for their role in clot formation, can exhibit altered functionality under pathological conditions such as cardiovascular diseases, metabolic disorders, and autoimmune diseases, impacting their interaction with coagulation factors and vascular endothelium. The review discusses the molecular and cellular mechanisms underlying platelet dysfunction, including aberrations in platelet activation, aggregation, and secretion. It also highlights the interplay between platelets and other components of the coagulation cascade, such as fibrinogen and clotting factors, in maintaining vascular integrity. Moreover, the review examines clinical implications, including how platelet dysfunction can be a contributing factor in conditions like deep vein thrombosis, stroke, and disseminated intravascular coagulation (DIC). Finally, current therapeutic approaches targeting platelet dysfunctions, including antiplatelet agents and emerging therapies, are reviewed to provide insights into potential strategies for managing fluid-coagulation system imbalances. This review underscores the importance of a comprehensive understanding of platelet dysfunction to improve diagnosis and treatment of hemostatic disorders.

## 1. Introduction

Platelets are essential for hemostasis, and their dysfunction can determine the increase in the bleeding risk, particularly in hematological disorders and thrombocytopenia, but also it may lead to an increased thrombotic risk. However, bleeding risk is not solely determined by platelet count; platelet function also plays a crucial role. Platelets adhere to injured vessels via von Willebrand factor and collagen receptors, triggering activation, shape change, and granule release. This leads to platelet aggregation through fibrinogen binding to GPIIb/IIIa receptors, forming a stable clot. Additionally, platelets support coagulation by exposing phosphatidylserine, which facilitates coagulation factor assembly and fibrin mesh formation, reinforcing clot stability.

Beyond hemostasis, platelets contribute to angiogenesis, inflammation, and tissue repair. They help maintain vascular integrity by filling endothelial gaps and preventing spontaneous bleeding, particularly in thrombocytopenic conditions. Inflammatory environments further highlight their role, as platelet receptors like GPVI and CLEC-2 are crucial for hemostasis in inflamed tissues. Platelet granule secretion also prevents tumor-associated bleeding by releasing factors involved in vasoconstriction and immune regulation. Despite being anucleate, platelets can synthesize proteins and transfer microparticles, influencing cellular responses. Their diverse functions suggest the potential for individualized therapeutic approaches based on disease context [1].

## 2. Literature Search

To explore the role of platelet dysfunctions in the pathogenesis of the hemostatic-coagulant system imbalances, a comprehensive literature search was conducted using databases such as PubMed, Scopus, and Web of Science. The search focused on studies published in peer-reviewed journals, with an emphasis on recent advancements in platelet biology, hemostatic disorders, and coagulation abnormalities. Keywords included “platelet dysfunction”, “coagulation imbalance”, “fluid homeostasis”, “hemostasis”, and “thrombocytopathy”. Articles discussing the molecular mechanisms of platelet activation, adhesion, and aggregation, as well as their interactions with coagulation factors and endothelial cells, were reviewed. Additionally, studies investigating the impact of platelet dysfunction in pathological conditions such as thrombocytopenia, thrombocytopathies, disseminated intravascular coagulation (DIC), and inflammatory states were included. Relevant clinical and experimental findings were assessed to provide a comprehensive understanding of how platelet abnormalities contribute to coagulation dysregulation, vascular instability, and fluid homeostasis disturbances.

## 3. Platelet Dynamics in Hemostasis: Activation, Aggregation, and Thrombus Formation

Platelets are essential mediators of hemostasis and thrombosis, circulating near vessel walls due to blood flow dynamics. This positioning enables their rapid response to vascular injury, where they adhere to the exposed extracellular matrix via receptors like glycoprotein VI and glycoprotein Ib-V-IX. Upon activation, platelets spread, aggregate, and release granules containing ADP, ATP, serotonin, and bioactive lipids, which amplify recruitment and clot formation. This process results in a thrombus with a densely packed core and a loosely bound outer shell, ensuring effective hemostasis.

**Figure 1:**
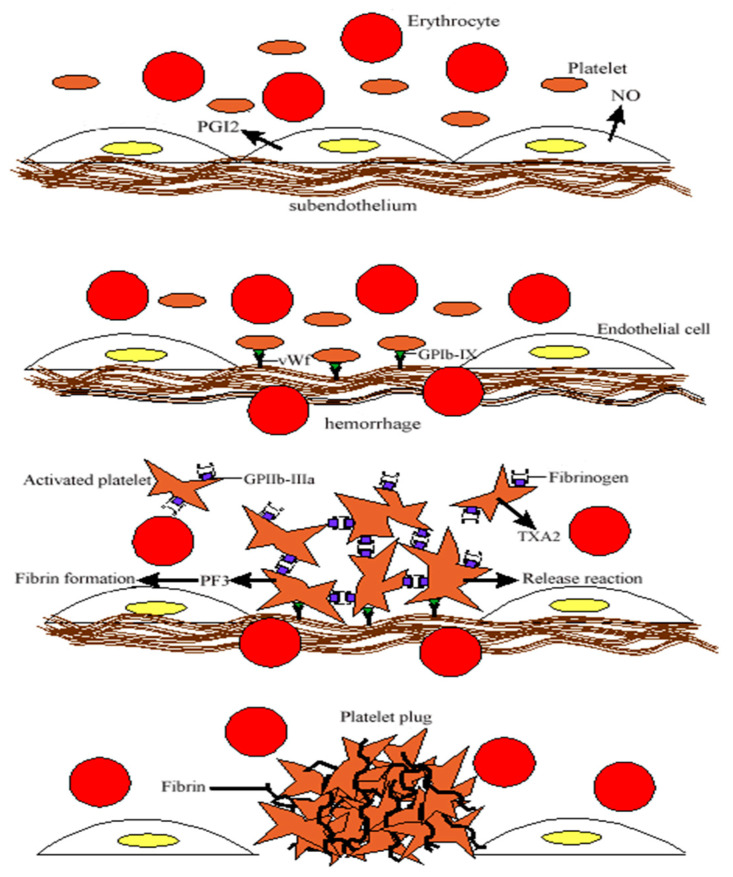
Schematic representation of primary hemostasis: platelet adhesion at the site of vascular injury, mediated by von Willebrand factor, which binds to GP Ib-IX receptors, followed by platelet aggregation through fibrinogen bridges bound to GP IIb-IIIa receptors, ultimately leading to the formation of the platelet thrombus.

Beyond hemostasis, platelets play a role in immunity and inflammation. Upon activation, they express adhesion molecules like P-selectin, which facilitate interactions with immune cells, promoting leukocyte recruitment and immune responses. Additionally, platelet surface receptors and Toll-like receptors contribute to pathogen recognition and immune signaling. Emerging evidence suggests platelets also influence tumor growth, metastasis, and intercellular communication via microparticles, highlighting their broader physiological significance [2] (Figure 1).

## 4. Platelets in Arterial and Venous Thrombosis: Mechanisms and Roles

Platelets play a pivotal role in the pathogenesis of both arterial and venous thrombosis, yet their contributions vary depending on the type of thrombosis and the underlying pathological conditions. While both conditions involve platelet activation, aggregation, and thrombus formation, the mechanisms driving platelet involvement differ in significant ways, particularly between high-shear arterial environments and the stasis-driven conditions of venous thrombosis.

- *Arterial Thrombosis: Initiation and Progression*

Arterial thrombosis primarily occurs following endothelial damage, which can be triggered by vascular injury or the rupture of atherosclerotic plaques, especially under high-shear stress conditions. When the endothelium is compromised, circulating platelets are rapidly decelerated and temporarily interact with exposed subendothelial matrix components, including immobilized von Willebrand factor (vWF) and collagen types I, III, and VI. The vWF–collagen interaction facilitates the initial tethering of platelets to the vessel wall through the glycoprotein Ib-V-IX complex. This interaction sets the stage for further platelet activation and adhesion [3,4].

Platelet receptors, such as GPVI and integrin α2β1, engage with fibrillar collagen in the subendothelial matrix, leading to a cascade of intracellular signaling events. This signaling activates integrin αIIbβ3, a key receptor for platelet aggregation. The active form of αIIbβ3 increases its affinity for adhesive proteins like vWF, fibrinogen, and fibronectin, promoting the formation of stable platelet aggregates. As platelets aggregate, they release and generate soluble agonists, such as ADP, thromboxane A2 (TxA2), and thrombin, which activate G protein-coupled receptors (GPCRs) on nearby platelets, driving additional platelet activation. This process culminates in the secretion of soluble factors, platelet spreading, and integrin activation, leading to the recruitment of more platelets and thrombus expansion [5].

- *Venous Thrombosis: Platelet Contributions and Microparticle Involvement*

Venous thromboembolism (VTE), encompassing deep vein thrombosis (DVT) and pulmonary embolism (PE), is characterized by Virchow’s Triad, which includes hypercoagulability, alterations in blood flow (stasis and turbulence), and endothelial dysfunction. Traditionally, platelets were considered less influential in venous thrombosis compared to arterial thrombosis. However, recent studies have highlighted their significant role in venous thrombus formation, especially in contexts such as cancer-associated thrombosis.

In venous thrombosis, platelet-derived microparticles (MPs) are a major contributor to thrombus formation, accounting for approximately 95% of the MPs found in circulation. Elevated levels of MPs have been observed in patients with VTE, particularly in those with cancer. In animal models, treatment with antiplatelet drugs like clopidogrel, a P2Y12 receptor inhibitor, has shown a reduction in tumor size and thrombus formation by preventing the accumulation of cancer cell-derived MPs. Additionally, the use of antiplatelet drugs, including aspirin (ASA), has been demonstrated to reduce venous thrombus formation in various animal models, further emphasizing the role of platelets in venous thrombosis [5,6].

Studies have also shown that the deletion of the P2Y1 receptor, a purinergic receptor expressed on platelets, reduces venous thrombosis formation, providing further evidence for the role of platelet signaling in this condition. Clinical trials, such as ASPIRE and WARFASA, have supported the use of ASA in preventing recurrent VTE after initial anticoagulation therapy. These trials demonstrated that ASA significantly reduces the recurrence of VTE by more than one-third without significantly increasing the risk of bleeding [7].

Therefore, we can say that platelets are essential mediators in both arterial and venous thrombosis, although their roles differ depending on the nature of the thrombotic event. In arterial thrombosis, platelets are activated through interactions with collagen and vWF, leading to integrin activation, platelet aggregation, and thrombus formation. In venous thrombosis, platelets contribute not only through direct aggregation but also by releasing microparticles that play a key role in thrombus development, particularly in conditions such as cancer. As research continues, the expanding understanding of platelet function in both arterial and venous thrombosis offers promising avenues for new therapeutic strategies targeting platelet activity, potentially improving patient outcomes in thrombotic diseases.

## 5. The Role of Platelets in Neoplasias

A long time ago, it was demonstrated that thrombocytopenic mice exhibited resistance to metastasis, marking the beginning of a deeper understanding of the relationship between platelets and cancer progression. Since then, numerous studies have affirmed the significant role of platelets in tumorigenesis. The connection between blood coagulation and cancer was first observed in the 1860s by French physician Armand Trousseau, who noted an association between venous thrombosis, blood hypercoagulability, and certain cancers, a phenomenon now known as Trousseau’s syndrome. Since then, various platelet receptors have been implicated in tumor progression, suggesting that therapies targeting platelets—typically used in cardiovascular disease—could have significant implications in cancer treatment [8].

Tumorigenesis is a complex, multi-step process that requires ongoing changes in both tumor cells and the tumor microenvironment (TME). Tumors need continuous growth signals, which they may either produce autonomously or receive from the surrounding TME. Platelet-derived microparticles have been shown to stimulate mitogen-activated protein kinases in lung carcinoma cells, promoting cell proliferation. Additionally, these microparticles increase the expression of matrix metalloproteinases (MMPs), enhancing tumor invasion and the remodeling of the extracellular matrix. By activating the same growth-promoting pathways seen in oncogenic mutations, platelets contribute significantly to tumor growth [9].

During tissue remodeling and inflammation, the balance between proliferation and apoptosis is tightly regulated. Tumor cells, however, must circumvent intrinsic programs that induce cell death to sustain their growth. Platelets play a role in this process by preventing apoptosis. They stimulate the expression of MMP-9 in colon and breast cancer cells, which promotes extracellular matrix remodeling, growth factor release, and reduced cell–cell contacts, all of which diminish apoptotic signals. Platelet microparticles can also inhibit intrinsic apoptosis by uncoupling mitochondria, independent of autophagy. These findings highlight the need for further investigation into how platelets influence both solid and hematologic cancers, particularly regarding cellular energetics and metabolism related to apoptosis.

Resistance to chemotherapy and molecularly targeted therapies remains a significant barrier to successful cancer treatment. Tumor cells develop mechanisms that promote resistance, including changes in integrin expression, the activation of oncogenic signaling, and the evasion of apoptotic signals. Platelet degranulation and activation create a growth factor-rich microenvironment that supports tumor proliferation and provides antiapoptotic benefits. Thrombocytosis (elevated platelet count) has been shown to increase chemoresistance, while thrombocytopenia (low platelet count) enhances chemotherapy efficacy. In murine breast cancer models, platelet depletion with antibodies increased the effectiveness of paclitaxel therapy by improving drug delivery to the tumor site. In clinical studies, elevated platelet counts in ovarian cancer patients were associated with poor prognosis and chemoresistance, further supporting the role of platelets in tumor progression and treatment resistance.

Tumor growth requires neovascularization to supply nutrients, remove waste, and oxygenate the tumor. While tumor cells were once thought to be the primary regulators of angiogenesis, it is now clear that the TME and stromal cells, including platelets, contribute significantly to this process. Platelets are a major source of proangiogenic factors, such as vascular endothelial growth factor (VEGF), platelet-derived growth factor (PDGF), and fibroblast growth factors (FGFs), all of which promote tumor growth and vascularization. Platelet-derived microparticles also release these factors, enhancing endothelial cell survival, proliferation, and vascularization in both healthy and diseased states. In patients with colorectal cancer, increased levels of platelet microparticles correlate with disease progression and metastasis, particularly in advanced stages [10,11].

Metastasis remains the leading cause of cancer-related death and presents a major challenge to improving patient outcomes. Platelets play a crucial role in facilitating metastasis by providing a “cloak” that shields circulating tumor cells from immune detection and mechanical damage in the bloodstream. This platelet “cloak” enables tumor cells to evade the immune system and protect themselves from shear forces in the vasculature, facilitating their extravasation at distant sites. Platelet interaction with tumor cells also primes them to adopt a mesenchymal invasive phenotype, promoting metastatic colonization. In breast cancer, platelet-derived lysophosphatidic acid (LPA) has been shown to stimulate metastatic growth by promoting osteoclast activation in the bone marrow, leading to bone destruction and further supporting metastatic growth.

The tumor is composed of a heterogeneous mix of cells, including a subpopulation of cancer stem cells with self-renewing capabilities. Stromal cells, including platelets, support these cancer stem cells, which are critical for tumor initiation, progression, and metastasis. Platelets cocultured with breast carcinoma cells have been shown to induce a cancer stem cell gene signature and promote epithelial-to-mesenchymal transition (EMT), further enhancing the tumor’s metastatic potential. High platelet counts correlate with poor prognosis in various cancers, underscoring the importance of platelets in supporting both tumor growth and the metastatic cascade [12].

In conclusion, platelets play multifaceted roles in cancer progression, from sustaining proliferative signals and evading cell death to enhancing angiogenesis, invasion, and metastasis. Their interactions with tumor cells in both the primary tumor and at metastatic sites underscore the potential therapeutic applications of platelet-targeting strategies in oncology. Understanding the complex signaling networks activated by platelets in the TME and their impact on tumor biology offers new avenues for improving cancer treatment and combating resistance to chemotherapy and targeted therapies. Future research is essential to fully elucidate the roles of platelets in cancer and to explore the therapeutic potential of cardiovascular inhibitors in oncology.

While the role of platelets in cancer progression, metastasis, and therapy resistance is well established, it is equally important to consider their involvement in primary hematologic disorders, such as essential thrombocythemia (ET). Unlike the reactive thrombocytosis observed in malignancies, where tumor-derived factors drive platelet production, ET represents a clonal proliferation of megakaryocytes due to intrinsic bone marrow pathology. Despite these distinct etiologies, both conditions share common mechanisms of platelet activation, thrombo-inflammatory complications, and vascular dysfunction. Given this overlap, the following section will explore ET and erythromelalgia, highlighting their pathophysiology and clinical implications while distinguishing them from cancer-associated platelet dysregulation.

- *Essential thrombocythemia (ET)*

Essential thrombocythemia is a monoclonal proliferation of hematopoietic stem cells characterized by an abnormal proliferation of megakaryocytes, leading to a persistently elevated platelet count in peripheral blood, often exceeding 600,000/mm^3^. The major clinical manifestations include hemorrhagic and/or thromboembolic diathesis [13].

Thrombotic manifestations are present at diagnosis in 20–45% of patients and can also occur during the disease course. They are a predominant feature of the disease and significantly more common than hemorrhagic manifestations. Thrombotic occlusions can be either arterial or venous, sometimes occurring simultaneously. They are more frequent in elderly individuals compared to younger patients with the same platelet count. Arterial thrombosis is present in 30% of patients at diagnosis. The most characteristic arterial thromboses result from platelet emboli or local platelet aggregation in small arteries, leading to occlusions that manifest as ischemia of the fingers, potentially progressing to necrosis or transient and established ischemic strokes. Thromboses may also affect larger arterial trunks, such as the axillary, carotid, or lower limb arteries, leading to intermittent claudication. Coronary artery thrombosis can cause anginal episodes and even myocardial infarction. Venous thrombosis is present in 6% of patients at diagnosis. Venous thrombosis affects both superficial and deep veins, predominantly in the limbs, but may also have unusual localizations that suggest an underlying myeloproliferative disorder. These include the splenic vein, portal vein, hepatic veins, mesenteric veins (causing abdominal pain), and renal veins. Spontaneous miscarriages are relatively common in essential thrombocythemia, caused by placental insufficiency due to the thrombosis of placental vessels [14].

- *Erythromelalgia*

Erythromelalgia is an almost pathognomonic sign but can also occur in patients with primary polycythemia who have significant thrombocytosis. It begins in the distal extremities of the lower limbs with acroparesthesias, followed by pain and a burning sensation in the feet, which become congested. These symptoms can be extremely painful and may be triggered by physical exertion or heat. Histological examination reveals arteriolar lumen narrowing due to endothelial cell swelling, smooth muscle cell proliferation, intracellular material deposition, and fragmentation of the internal elastic lamina. These changes result from platelet-mediated acral inflammation and arteriolar thrombosis [15] (Table 1).

## 6. Bleeding Caused by Congenital Platelet Disorders

***Glanzmann thrombasthenia (GT)*** is an autosomal recessive bleeding disorder affecting the megakaryocyte lineage, characterized by impaired platelet aggregation. It is a moderate to severe hemorrhagic condition with predominant mucocutaneous bleeding. The molecular cause involves abnormalities in the αIIbβ3 integrin, which is essential for platelet aggregation at injury sites.

First described by Glanzmann in 1918 as “hereditary hemorrhagic thrombasthenia”, early diagnostic indicators included prolonged bleeding time and a scattered platelet appearance on blood smears. In 1956, Braunsteiner and Pakesch highlighted the condition as an inherited disorder where platelets fail to spread or support clot retraction. In 1964, Caen et al. clarified that the key diagnostic feature is the absence of platelet aggregation. Patients were classified into type I (no aggregation or clot retraction), type II (no aggregation, with clot retraction), and variant disease (first defined in 1987). Early reports highlighted the clinical variability of Glanzmann thrombasthenia (GT): some patients experience minimal bruising, while others suffer from severe, potentially fatal hemorrhages. Hemorrhagic symptoms occur only in homozygous individuals, with heterozygous carriers typically being asymptomatic despite having half-normal platelet αIIbβ3 levels. Common bleeding sites include purpura, epistaxis, gingival bleeding, and menorrhagia, while gastrointestinal bleeding and hematuria, though less frequent, can lead to serious complications. Deep visceral hematomas, seen in coagulation disorders like hemophilia, are rare in GT. Bleeding symptoms usually appear early in life, often with more severe epistaxis in childhood, but tend to decrease with age. Co-existing inherited conditions, such as mild von Willebrand disease, may worsen bleeding severity [16].

While GT can be severe, the prognosis is generally excellent with proper care. Most adults live in good health, with minimal daily impact from the condition. Death from hemorrhage is rare, typically occurring only with trauma or other diseases like cancer. However, families often report deceased siblings upon the diagnosis of GT. Clinical observations suggest that there is little correlation between the amount of residual platelet αIIbβ3 and the severity of bleeding in GT. Some patients with virtually no detectable αIIbβ3 experience minimal symptoms, while others with 10–15% of normal αIIbβ3 levels may have severe hemorrhages. The absence of αvβ3 in vascular cells may contribute to bleeding, but even in families with undetectable β3, symptoms vary widely. While platelet αIIbβ3 inhibition protects against arterial thrombosis, GT patients may also be protected from atherosclerotic disease, although studies suggest otherwise. They are not protected from venous thrombosis.

Mice lacking β3 integrins show thickened bones due to dysfunctional osteoclasts, but in some human cases, the compensatory upregulation of α2β1 integrin may prevent this effect. β3 knockout mice also display increased vascular EGF signaling and enhanced angiogenesis, which may influence tumor growth and fertility, topics requiring further research in GT patients with β3 defects [17,18].

***Bernard–Soulier syndrome (BSS****)*, or hemorrhagiparous thrombolytic dystrophy, is a rare inherited bleeding disorder marked by giant platelets, thrombocytopenia, and prolonged bleeding. First described in 1948 by Jean-Bernard and Jean-Pierre Soulier, BSS results from defects in the GPIb-IX-V complex, a key platelet receptor complex that binds to von Willebrand factor (vWF) and plays a crucial role in thrombosis and hemostasis.

BSS is caused by mutations in genes encoding GPIb-alpha (GPIBA), GPIB-beta (GPIBB), and GPIX (GP9), which are essential for the GPIb-IX-V complex. These mutations, often inherited in an autosomal recessive pattern, are primarily homozygous and linked to consanguinity. Some families carry asymptomatic carriers, and autosomal dominant inheritance is rare. Mutations are found mainly in the GP1BA (28%), GP1BB (28%), or GP9 (44%) genes, with various genetic alterations such as nonsense, missense, frameshift, and deletion/insertion mutations. BSS can be biallelic (biallelic BSS) or monoallelic (monoallelic BSS), with the former leading to more severe symptoms. Mutations have been identified globally, with notable examples from China, Denmark, Reunion Island, and the Czech Republic. The GPIb-IX-V complex, located on the platelet surface, plays a crucial role in platelet adhesion to the subendothelium, initiating clot formation when the vascular subendothelium is exposed or a plaque ruptures. It is also involved in deep venous thrombosis (DVT) [19].

The primary function of the GPIb-IX-V complex is to bind with von Willebrand factor (VWF), triggering a signaling cascade that activates the platelet integrin GPIIb-IIIa and promotes platelet aggregation. While VWF is a weak agonist, full platelet activation requires additional signaling through thromboxane A2 and ADP pathways.

The N-terminal of GPIb-alpha is vital for platelet coagulation, as it binds to high molecular weight kininogen, Factors XI and XII, and alpha-thrombin. It also serves as a key site for interactions between platelets and leukocytes in thrombosis and inflammation.

Additionally, the GPIb-IX-V complex helps maintain platelet shape by connecting the platelet surface to the actin filaments of the membrane skeleton. This is mediated by specific regions of the GPIb-alpha cytoplasmic tail, which binds to filamin A, an actin-associated protein.

Given these essential functions, mutations in the genes encoding the GPIb-IX-V complex can impair platelet activation and adhesion, leading to reduced clot formation and the presence of giant platelets, a hallmark of Bernard–Soulier syndrome (BSS) [20].

***Von Willebrand disease (vWD)*** is the most common inherited bleeding disorder, characterized by mucocutaneous bleeding that can significantly affect quality of life. Despite its prevalence and morbidity, diagnosing and classifying vWD remains challenging, largely due to the wide variability of plasma VWF levels in the general population and VWF’s multiple functions in the body. Recent advancements have clarified VWF’s biological roles and the underlying pathophysiology of both quantitative and qualitative forms of vWD. New laboratory tests now enable the more precise evaluation of VWF activity. This review discusses these advances in vWD diagnosis and how they may improve clinical diagnostic algorithms. It also covers recent progress in treatment options for vWD patients. vWD can result from either a quantitative or qualitative VWF deficiency and is categorized into types based on the defect. Types 1 and 3 are quantitative defects, with Type 1 being a partial deficiency and Type 3 being an absolute deficiency. Type 1 includes subtype 1C, characterized by increased VWF clearance. Type 2 involves qualitative defects and includes subtypes 2A (multimerization defect), 2B (increased binding to GPIb), 2N (defective FVIII binding), and 2M (impaired binding with normal multimers).

Type IIB von Willebrand Disease (vWD) shares a clinical phenotype similar to Bernard–Soulier syndrome (BSS). In type IIB vWD, the increased affinity of large VWF multimers for platelets causes rapid platelet clearance, resulting in thrombocytopenia. Additionally, the platelets in type IIB vWD are enlarged, but they exhibit increased aggregation in response to ristocetin. In contrast, patients with BSS show low to absent platelet aggregation in response to ristocetin. Moreover, platelet-type vWD is associated with mutations in the GPIb-alpha gene, which enhance the platelets’ affinity for VWF [21].

***Paris-Trousseau syndrome (PTS)*** is a rare inherited thrombocytopenic disorder reported in fewer than a dozen patients and caused by a deletion on the long arm of chromosome 11. Affected individuals have a normal platelet lifespan in circulation but a significant increase in megakaryocytes in the bone marrow. However, megakaryocyte maturation is arrested, and many cells fail to progress to terminal differentiation stages, impairing platelet release. Megakaryocyte platelet release involves a unique process where the cytoplasm forms beaded projections (proplatelets), which transport organelles along microtubules to assemble new platelets. Research using thrombopoietin to expand megakaryocytes and mouse models of thrombocytopenia has accelerated the understanding of platelet biogenesis. In this issue, Raslova et al. discuss the transient monoallelic expression of a transcription factor gene involved in platelet release, explaining why PTS patients with one normal chromosome 11 copy produce few, defective platelets. Similar regulatory mechanisms may underlie other dominant genetic disorders where haploinsufficiency or interference with the wild-type gene does not fully explain the condition. As said before, it is a congenital platelet disorder commonly found in patients with Jacobsen syndrome, a rare inherited condition characterized by skull dysmorphism, developmental delay, and multiple organ abnormalities. PTS features a mild lifelong bleeding tendency, macrothrombocytopenia, bone marrow dysmegakaryopoiesis, and giant fused α-granules in a small subset of platelets. It is caused by a terminal deletion on the long arm of chromosome 11 (11q23.3), which contains the genes ETS1 and FLI1, located at 11q24.3. The role of hemizygous deletion of one or both of these genes in PTS is still uncertain [22].

In a 2004 study, Raslova et al. suggested that the platelet disorder in PTS is due to the allelic exclusion of FLI1 in CD41+/CD42− progenitors, affecting only the megakaryocyte subpopulation lacking FLI1. More recent findings implicate missense mutations and deletions in the DNA-binding domain of FLI1 as a probable cause of the megakaryocyte and platelet defects in PTS patients. While FLI1 hemizygous deletion is likely a key factor in inherited thrombocytopenia, the detailed platelet defect in PTS has yet to be fully compared. Mice lacking FLI1 experience embryonic hemorrhage, related to its role in megakaryopoiesis, endothelium, and hemangioblast specification. However, hemizygous FLI1 deletion in mice does not fully replicate the human PTS phenotype [23,24].

***Quebec platelet disease (QPD)*** is an autosomal dominant bleeding disorder characterized by a platelet-dependent, gain-of-function defect in fibrinolysis, without affecting systemic fibrinolysis. The disorder is marked by a more than 100-fold overexpression of PLAU, specifically in megakaryocytes. This overproduction leads to a similar increase in platelet stores of urokinase plasminogen activator (PLAU/uPA), which, in turn, accelerates fibrinolysis through plasmin-mediated degradation of various α-granule proteins. The mutation responsible for QPD is a 78-kb tandem duplication of PLAU, though the mechanism behind megakaryocyte-specific PLAU overexpression remains unclear. Liang et al. described the functional mechanism behind the megakaryocyte-specific increase in PLAU expression and platelet-dependent fibrinolysis in Quebec platelet disorder (QPD). A decade after the discovery that QPD is caused by tandem duplication, Liang et al. showed that this duplication places an extra copy of PLAU on the opposite side of a CTCF genomic boundary. As a result, the extra PLAU copy is no longer insulated from one of the transcriptional enhancers of the VCL gene, leading to elevated transcription levels. This process, known as “enhancer hijacking”, was first observed in Burkitt lymphoma nearly 40 years ago. Though additional examples have since been found, diagnosing new occurrences remains challenging and time-consuming [25,26].

***Hermansky–Pudlak syndrome (HPS)*** is a rare autosomal recessive disorder characterized by highly penetrant pulmonary fibrosis in young adults, particularly in subtypes HPS-1, HPS-2, and HPS-4. Other hallmark clinical features include oculocutaneous albinism and bleeding diathesis, which facilitate early identification of at-risk individuals before lung disease manifests.

HPS is an extremely rare genetic disorder with ten identified subtypes. It has been reported in diverse populations across the world, including Western Europe, the Indian subcontinent, Japan, China, the Middle East, and among non-Puerto Rican Hispanic groups. As of now, over 1200 individuals have registered with The HPS Network, Inc., a nonprofit organization supporting those affected by the condition. The exact prevalence of HPS and its subtypes remains unknown. In northwest Puerto Rico, the founder mutation (a 16 bp duplication in exon 15 of HPS1) is carried by approximately 1 in 21 individuals, with an estimated disease frequency of 1 in 1800 for HPS-1. Another founder mutation (a 3904 bp deletion in HPS3) results in HPS-3 affecting 1 in 4000 individuals in central Puerto Rico. HPS-1 is the most prevalent subtype among non-Puerto Rican individuals, accounting for approximately 25% of cases. Reports of other subtypes, particularly HPS-3, have also been documented in individuals of Ashkenazi Jewish descent [27].

The ten genetically distinct subtypes of HPS (HPS-1 through HPS-10) share key features such as oculocutaneous albinism and platelet storage pool deficiency. The gene products associated with HPS are expressed ubiquitously and form hetero-oligomeric complexes called BLOCs (biogenesis of lysosome-related organelle complexes), which play a crucial role in trafficking to lysosome-related organelles, including melanosomes and platelet dense granules.

- **HPS1 mutations** disrupt BLOC-3 function and are strongly linked to the development of pulmonary fibrosis.
- **HPS3 mutations** affect BLOC-2, a different trafficking complex, and are not associated with pulmonary fibrosis.
- **HPS2 mutations** impact the AP-3 complex, a hetero-oligomeric protein involved in early endosomal protein trafficking.

Emerging research suggests that HPS pathogenesis involves macrophage-driven inflammation preceding pulmonary fibrosis. Bronchoalveolar lavage (BAL) fluid from HPS patients shows an increased number of activated macrophages that secrete excessive cytokines and chemokines. Studies also indicate fibroblast abnormalities, including elevated galectin-3 expression, a molecule with profibrotic properties. Additionally, patients with HPS-related pulmonary fibrosis (HPS-PF) have increased circulating CXCR4-positive fibrocytes compared to those without lung disease [28].

Due to the limited availability of human lung tissue, HPS research has relied heavily on murine models. HPS mouse models closely resemble human disease phenotypes, including hypopigmentation, platelet granule deficiency, and genotype-specific macrophage activation. While spontaneous fibrosis does not occur naturally in HPS mice, models of HPS-1 and HPS-2 exhibit exaggerated fibrotic responses to stimuli such as silica and bleomycin. Notably, a double-mutant HPS-1/2 model has demonstrated spontaneous pulmonary fibrosis (Table 2).

Macrophage-mediated inflammation is also evident in HPS mouse models. However, bone marrow transplant studies suggest that fibrosis susceptibility stems from alveolar epithelial dysfunction rather than intrinsic macrophage defects. Additional studies point to alveolar epithelial apoptosis, IL-13Rα2 signaling, and autophagy dysregulation as potential mechanisms contributing to disease progression. Despite these insights, the precise link between HPS-related trafficking defects and alveolar epithelial dysfunction leading to fibrosis remains an area of active investigation. The bleeding disorder in HPS arises from the absence of dense bodies (δ granules) in platelets, despite a normal platelet count. Platelet storage granules (α and δ granules) play a crucial role in hemostasis by releasing their contents (ATP, ADP, serotonin) to recruit additional platelets following the initiation of the aggregation cascade. Historically, bleeding time was used to assess platelet aggregation defects; however, it has been found to be unreliable and is no longer recommended. The most accurate diagnostic method remains electron microscopy analysis of freshly isolated plasma, which confirms the complete or near-complete absence of δ granules [29].

***Chediak–Higashi syndrome (CHS)*** is a rare autosomal recessive disorder characterized by congenital immunodeficiency, bleeding diathesis, recurrent pyogenic infections, partial oculocutaneous albinism, and progressive neurodegeneration. The condition is also associated with hemophagocytic lymphohistiocytosis (HLH), also known as the “accelerated phase”, a severe hyperinflammatory state that is the leading cause of mortality in CHS.

A hallmark diagnostic feature of CHS is the presence of enlarged granules within leukocytes on a peripheral blood smear. Neutropenia, along with reduced Natural Killer (NK) cell numbers and function, may also be observed. Fewer than 500 cases have been documented worldwide.

CHS is caused by biallelic mutations in the LYST gene, located at 1q42.3 on chromosome 1, spanning approximately 222 MB of DNA. Most LYST mutations are homozygous or compound heterozygous and include missense, frameshift, or nonsense variants. LYST encodes a large protein involved in lysosomal trafficking, though its precise function remains unclear despite being identified as the causative gene over six decades ago.

Patients with CHS have an increased bleeding tendency, despite typically normal platelet counts. This is due to a deficiency or absence of platelet-dense granules, leading to coagulation defects that manifest as easy bruising and mucosal bleeding.

Historically, CHS has been classified as “typical” or “atypical” based on clinical presentation. However, natural history studies suggest that this distinction is arbitrary, as both groups exhibit overlapping and highly variable phenotypes.

Most individuals with CHS develop hemophagocytic lymphohistiocytosis (HLH), a potentially fatal inflammatory disorder characterized by fever, cytopenias, hepatosplenomegaly, and lymphadenopathy. HLH in CHS has been linked to defective NK cell function and Epstein–Barr virus infection. Initial treatment typically involves corticosteroids and chemotherapy, followed by hematopoietic stem cell transplantation (HSCT), which is often pursued soon after diagnosis to correct immunologic and hematologic abnormalities. Patients with the atypical form of CHS appear to have a lower risk of developing HLH [30].

***Thrombocytopenia with absent radii (TAR) syndrome*** is a rare autosomal recessive disorder characterized by hypomegakaryocytic thrombocytopenia and bilateral radial aplasia, affecting skeletal, hematologic, and cardiac systems. Some researchers suggest this association arises from the simultaneous development of the heart, radii, and megakaryocytes during weeks 6–8 of gestation.

Thrombocytopenia typically manifests at birth or in the neonatal period but may recur later. The disease’s low prevalence and limited bone marrow samples have hindered studies on its pathophysiology. The available data suggest thrombocytopenia results from a decreased response to thrombopoietin (TPO), affecting both the proliferation and differentiation of megakaryocytes. Genetic studies have ruled out c-mpl and TGF-β2 mutations as primary causes.

Colony growth studies in TAR patients have produced mixed results, ranging from no megakaryocyte (MK) colony growth to reduced colony size and cell numbers, suggesting a defective TPO signaling pathway. However, granulopoiesis and erythropoiesis remain unaffected. More research is needed to clarify TAR syndrome’s molecular mechanisms [31].

***Wiskott–Aldrich syndrome (WAS)*** is a rare X-linked primary immunodeficiency disorder with an incidence ranging from 1 in 50,000 to 1 in 250,000 live births. In its classic form, WAS presents early in life with a triad of recurrent infections, eczema, and microthrombocytopenia, caused by loss-of-function mutations in the WAS gene. WAS is expressed exclusively in hematopoietic cells and encodes the Wiskott–Aldrich syndrome protein (WASp), a critical regulator of the actin cytoskeleton that coordinates actin filament assembly in response to cell signaling events. Defects in WASp function impair processes in both myeloid and lymphoid cells, including cell adhesion, migration, phagocytosis, immune synapse formation, and more recently, autophagy and inflammasome regulation. The platelet defect in WAS is only partially understood, likely due to a combination of megakaryocyte dysfunction leading to abnormally small or malformed platelets and increased platelet destruction in the spleen. Despite this, megakaryocyte numbers in the bone marrow are typically normal.

The early recognition of WAS is critical, as curative stem cell and gene therapies are available. Without treatment, the median survival is reduced to 10–15 years due to infections, severe bleeding, autoimmune complications, and hematological malignancies. Milder forms of the disorder, such as X-linked thrombocytopenia (XLT), present with a similar bleeding phenotype but lack other significant clinical features, and can generally be managed conservatively.

Wiskott–Aldrich syndrome protein (WASp) plays a crucial role in both innate and adaptive immunity by regulating actin cytoskeleton-dependent processes such as immune synapse formation, cell signaling, migration, and cytokine release. Recent studies also suggest that WASp may be directly involved in nuclear transcription programs, independent of actin polymerization. WASp is widely expressed in non-erythroid hematopoietic cells. Since the identification of the WAS gene over 20 years ago, around 300 mutations have been described, leading to a wide range of clinical manifestations, including immunodeficiency, inflammation, bleeding disorders, autoimmunity, and potential malignancies. Recent reviews have discussed the clinical aspects and emerging treatments of WAS in detail [32].

WASp is a 502-amino acid cytosolic protein with several distinct domains: an Ena-VASP homology (EVH1, or WH1) domain at the N-terminus, a basic domain (B), a guanosine triphosphatase-binding domain (GBD), a polyproline (PP) domain, and a verprolin homology/central/acidic (VCA) domain at the C-terminus. As a key regulator of the actin cytoskeleton in hematopoietic cells, WASp plays vital roles in lymphoid and myeloid cell migration, receptor signaling, cytotoxicity, and phagocytosis. Additionally, cytoskeleton-independent roles of WASp in nuclear transcription have recently been identified.

In its resting state, WASp is autoinhibited, with the VCA domain bound to a hydrophobic pocket in the GBD domain. Upon binding with partners such as the Rho family GTPase CDC42 or phosphorylation of a tyrosine residue (Y291 in human WASp), the autoinhibited conformation is destabilized, exposing the VCA region. This allows for ARP 2/3 binding, leading to actin filament nucleation and branching. Other proteins, such as the NCK adaptor and various tyrosine kinases, also promote WASp activation. The phosphorylation of two serine residues in the VCA domain enhances the affinity for ARP 2/3, further regulating WASp activation. Evidence suggests that WASp activity can also be modulated by oligomerization, with active WASp forming complexes that more effectively stimulate ARP 2/3. Furthermore, the phosphorylation of Y291 marks WASp for degradation by calpain and the proteasome. WASp-interacting protein (WIP) stabilizes WASp by binding to the EVH1 domain, protecting it from degradation, and aiding its localization to sites of actin polymerization. Microthrombocytopenia is a strong indicator of WAS, but genetic analysis is essential for definitive diagnosis, guiding management, and family screening. Over 300 mutations have been identified, with new cases emerging as genetic testing becomes more accessible. Mutations occur throughout the gene, with missense mutations commonly clustered in the first four exons [33].

Genetic details alone do not always predict clinical severity, but combining mutation information with WASp levels helps establish a genotype-phenotype correlation. Therefore, we analyze WASp expression as part of the diagnostic process. Flow cytometry has replaced Western blotting for WASp quantification due to its speed and reliability. However, protein expression alone is not always definitive, as missense mutations can preserve normal levels of impaired WASp, and absent expression may result from epitope detection issues [34,35]. Collagen receptor deficiencies, including defects in GPVI and α2β1, lead to impaired platelet adhesion and activation, compromising primary hemostasis. Similarly, alpha granule deficiency, also known as gray platelet syndrome, results in defective secretion of essential procoagulant factors, further disrupting clot formation. In cases of congenital afibrinogenemia, the complete absence of fibrinogen prevents platelet aggregation, making hemostatic plug formation ineffective. P2Y12 receptor deficiency impairs platelet response to ADP, a crucial mediator of aggregation, leading to a bleeding tendency. Additionally, cyclooxygenase-1 (COX-1) deficiency affects thromboxane A2 synthesis, a key factor in platelet activation and recruitment, further impairing platelet function and contributing to an increased risk of bleeding [36] (Table 3).

## 7. Bleeding in Acquired Platelet Function Disorders

*Acquired platelet function disorders (PFDs)* are a diverse group of conditions where platelet dysfunction leads to moderate-to-severe bleeding. These disorders should be suspected in patients with unexplained mucocutaneous bleeding of recent onset, no prior history of hemorrhages, and normal coagulation tests and platelet counts. Drug-induced PFDs are the most common and can often be traced to recent use of platelet-inhibiting medications. Occasionally, platelet dysfunction may be linked to medications or supplements that affect platelet function non-specifically. Autoimmune PFDs can also occur, where autoantibodies target platelet receptors, causing dysfunction. This is suspected in patients with mild/moderate thrombocytopenia and bleeding that is disproportionate to platelet count. Autoimmune PFDs are often associated with autoimmune or lymphoproliferative diseases, but primary cases have also been reported. Bleeding symptoms may appear before or alongside the primary disease, which can lead to missed diagnoses due to the rarity of autoimmune PFD. Such disorders should be suspected when there is unexplained bleeding, no antiplatelet drug use, normal coagulation tests, and normal or slightly reduced platelet counts [37,38].

Symptoms of acquired platelet function disorders (aPFDs) are typically associated with platelet dysfunction and range from mild to severe spontaneous mucocutaneous bleeding. These include ecchymosis, hematomas, petechiae, nose and gum bleeding, upper and lower gastrointestinal bleeding, menorrhagia, subconjunctival hemorrhage, and hematuria. Intracranial hemorrhage, the most severe complication of platelet disorders, is rare in aPFDs. However, one case involved a patient with a δSPD associated with chronic lymphocytic leukemia (CLL), who experienced three episodes of subdural hematoma as initial symptoms of CLL. Despite a significant response to CLL therapy, bleeding symptoms persisted until rituximab therapy, which improved platelet dysfunction and platelet count, leading to the resolution of symptoms. The early recognition of acquired platelet dysfunction is essential to prevent and manage severe bleeding complications. Other reported symptoms include bleeding after surgery or trauma, such as intraocular bleeding during eye surgery, epidural hematoma following intrathecal injection, excessive bleeding after tonsillectomy or tooth extraction, post-trauma elbow hemarthrosis, and significant bleeding during surgical repair of an enterocutaneous fistula. Autoimmune platelet function disorders (aPFDs) can be primary or secondary to underlying conditions, such as hematological malignancies or immune diseases. Secondary forms often involve multiple contributing conditions [39,40].

***Primary and secondary acquired Glanzmann thrombasthenia (aGT):*** Of the reported aGT cases, 9 were primary with no other diseases, while 18 were linked to lymphoproliferative diseases. These included Hodgkin lymphoma, non-Hodgkin lymphoma, acute lymphoblastic leukemia, hairy cell leukemia, multiple myeloma, and monoclonal gammopathy. Nine cases had concurrent autoimmune disorders, such as systemic lupus erythematosus, autoimmune hemolytic anemia, and acquired von Willebrand disease. Additionally, four cases were associated with organ transplants and immunosuppressive therapies, with tacrolimus being particularly controversial. In some cases, aGT was triggered by drugs like diclofenac, with recovery after discontinuation [40].

*Immune-mediated thrombotic thrombocytopenic purpura (iTTP)* is an acute thrombotic microangiopathy caused by an acquired, antibody-mediated deficiency of the ADAMTS13 protease. Recent advancements have shifted the focus toward improving recovery from acute episodes and managing long-term complications. iTTP is now recognized as a chronic disease with lasting effects, including cardiovascular and neurocognitive impairments, which may contribute to reduced survival in affected individuals [41].

***Primary and secondary acquired delta storage pool disease (aδSPD):*** Among aδSPD cases, autoimmune and connective tissue disorders were most commonly reported, with five cases linked to lupus, rheumatoid arthritis, or unspecified connective tissue diseases, and three linked to lymphoproliferative disorders. Only one case was primary. Some cases were associated with chronic immune thrombocytopenic purpura (ITP) [37].

***Other autoimmune platelet function disorders:*** In cases with mixed or uncertain diagnoses, one study linked hairy cell leukemia to aδSPD with no platelet-associated IgG, suggesting direct platelet activation by hairy cells. Other cases involved storage pool deficiency or a combination of congenital and acquired defects. A patient with chronic lymphocytic leukemia and other autoimmune conditions exhibited a normal platelet count but still had bleeding symptoms, later confirmed as aGT.

A variety of immune-mediated mechanisms are implicated in these disorders, with some cases tied to chronic lymphocytic leukemia, systemic lupus erythematosus, and other autoimmune conditions [38].

***Drug-mediated thrombocytopenia*** can be classified into immune-mediated and nonimmune-mediated types, each with distinct pathophysiological mechanisms.

Nonimmune drug-induced thrombocytopenia (DITP) occurs due to the direct cytotoxic effects of certain drugs on megakaryocytes or platelets, leading to impaired thrombopoiesis or increased platelet destruction. This is commonly observed with antineoplastic agents, which are toxic to hematopoietic stem cells, and with the antibiotic linezolid, which has been associated with myelosuppression. Additionally, some drugs, such as oxaliplatin, induce severe thrombocytopenia through drug-induced platelet antibodies rather than direct cytotoxicity. Certain medications may also trigger platelet apoptosis via Ca^2+^ signaling, mitochondrial depolarization, and phosphatidylserine exposure, though the clinical significance of this mechanism remains unclear. More than 300 drugs have been implicated in DITP, with frequently reported culprits including quinine, vancomycin, carbamazepine, ceftriaxone, rifampin, and heparin [39,40].

Immune-mediated DITP involves drug-dependent antibodies (DDAbs) that target platelets through various mechanisms:

1. Quinine-type DDAbs—These bind to GPIIb/IIIa or GPIb/IX only in the presence of the drug, forming immune complexes that trigger platelet destruction.
2. Hapten-dependent DDAbs—Small molecules (e.g., penicillin) bind covalently to platelet proteins, eliciting an immune response.
3. Fiban-type DDAbs—Antibodies recognize structural changes in GPIIb/IIIa induced by fiban-type platelet inhibitors.
4. Drug-specific DDAbs—Target drugs containing murine components (e.g., abciximab), leading to immune-mediated platelet clearance.
5. Autoantibody mechanism—Drug-induced antibodies, such as those from gold therapy, persistently bind to platelets even in the absence of the drug.
6. Immune complex formation—Some DDAbs form immune complexes that activate platelets via Fcγ receptors, further exacerbating thrombocytopenia.

Understanding the diverse mechanisms underlying drug-mediated thrombocytopenia is crucial for accurate diagnosis and effective management. Further research is needed to refine detection methods and explore targeted treatment strategies [42].

***Uremia***, a condition resulting from chronic kidney disease (CKD), leads to platelet dysfunction primarily due to the accumulation of uremic toxins that impair platelet adhesion, activation, and aggregation. These toxins disrupt von Willebrand factor (vWF) interactions with platelets, reduce thromboxane A2 synthesis, and alter glycoprotein receptor expression, leading to an increased risk of bleeding despite normal platelet counts. Additionally, nitric oxide (NO) and prostacyclin levels are elevated in uremia, further inhibiting platelet function. Clinical manifestations of uremic platelet dysfunction include prolonged bleeding time, easy bruising, and mucosal bleeding. Management strategies focus on dialysis to reduce toxin levels, administration of desmopressin (DDAVP) to enhance vWF release, and use of conjugated estrogens or platelet transfusions in severe cases [43].

***Myeloproliferative neoplasms (MPNs)***, including essential thrombocythemia (ET), polycythemia vera (PV), and primary myelofibrosis (PMF), are associated with both thrombotic and bleeding complications due to dysfunctional platelet production and activity. In MPNs, platelet counts may be elevated, but the platelets often exhibit impaired aggregation due to qualitative defects, such as abnormal granule content, defective receptor expression, and dysregulated signaling pathways. The presence of the JAK2 V617F mutation, common in MPNs, further promotes a prothrombotic state by increasing platelet reactivity and endothelial dysfunction. Patients with MPN-associated platelet disorders are at risk of paradoxical thrombosis (e.g., stroke, deep vein thrombosis) and hemorrhagic events (e.g., gastrointestinal or mucosal bleeding). Treatment strategies involve cytoreductive therapy (e.g., hydroxyurea or interferon-α) to control platelet counts, low-dose aspirin for thrombosis prevention, and careful monitoring to balance the risk of bleeding and clotting [44].

***In cardiopulmonary bypass (CPB)***, thrombocytopenia occurs due to multiple mechanisms. Platelet activation is triggered by adhesion and aggregation on fibrin deposits within the extracorporeal circuit, followed by mechanical platelet destruction. Additionally, inflammatory processes stimulate the release of cytokines, which can lead to platelet dysfunction and destruction, further contributing to thrombocytopenia. After bypass, platelets may be temporarily sequestered in the spleen and lungs, further justifying the decrease in platelet count.

Typically, the reduction in platelet count during CPB is transient, with platelet levels increasing again in the days following surgery [45].

Platelet dysfunction can also occur in other types of surgical procedures (as outlined in Table 4), particularly those involving significant blood loss, extracorporeal circulation, or predominant systemic inflammation. In these types of surgeries, platelet dysfunction may be caused by factors such as hypothermia, hemodilution, systemic inflammation, heparin use, disseminated intravascular coagulation (DIC), and dysfunction of organs involved in platelet production or regulation, including the bone marrow, spleen, and kidneys [46].

***Diet*** can influence platelet aggregation, leading to an increased risk of bleeding or thrombosis. There are several nutrients that promote platelet aggregation and increase thrombotic risk. These include saturated fatty acids (which stimulate inflammatory processes, leading to secondary platelet activation), refined sugar, ultraprocessed foods (excess sugar can increase oxidative stress, leading to platelet hyperreactivity), and excessive alcohol (which causes endothelial dysfunction, promoting hypercoagulable phenomena). On the other hand, deficiencies in certain vitamins or trace elements can lead to changes in platelet function: deficiencies in vitamin B12 and folic acid, or iron deficiency, lead to the development of anemic syndromes involving secondary platelet dysfunction, while vitamin C deficiency affects the integrity of the vascular endothelium and increases the risk of bleeding [46].

***Chronic liver diseases*** are often associated with significant impairment of platelet function, which is clinically evident. In addition to thrombocytopenia induced by hypersplenism, the following are also observed: reduced platelet adhesiveness, abnormal aggregation induced by ADP, adrenaline, and thrombin, and a decrease in the availability of platelet factor 3. All of these mechanisms may be due to the toxic effect of alcohol on bone marrow megakaryocytes. Additionally, primary activation of the fibrinolytic system occurs, with increased circulating PDF that alters platelet function. Fragments D and E have a higher affinity for the platelet membrane, binding to it and interfering with its functions [47].

## 8. Platelets: A Bridge Between Hemostasis and Immunity

It is believed that platelets and leukocytes share a common ancestral cell, the thrombocyte, which allows them to perform both hemostatic and immune functions in lower vertebrates, such as fish and birds. This connection explains why platelets are frequently involved in immunological functions. While not traditionally part of the inflammatory pathway, platelets can be seen as an extension of the cellular immune system. Recent findings place platelets at the center of various inflammatory processes, influencing both normal leukocyte biology and inflammatory signals. Several key observations support the view of platelets as immune cells. For instance, the expression of Toll-like receptors (TLRs) on platelets suggests that they can directly interact with microbial pathogens, much like leukocytes. Since only a small fraction of platelets are required to maintain adequate hemostasis, the larger population serves as significant vascular surveillants, identifying foreign particles in the bloodstream.

Emerging evidence also highlights platelets’ role in cardiovascular disease development, particularly in chronic inflammation, which is a key driver of atherosclerosis. Platelets adhere to von Willebrand factor (VWF) bound to endothelial cells, initiating the tethering and rolling of leukocytes on the endothelial surface. In this way, platelets not only bring leukocytes to areas of inflammation that could lead to atherosclerosis but also store proinflammatory mediators such as thromboxanes and the CD40 ligand. Additionally, platelets activate the complement system, and complement proteins, in turn, activate platelets. In experimental models, hyperlipidemia has been shown to recruit platelets to the endothelial layer, with VWF, glycoprotein Ib-IX, and P-selectin playing crucial roles in this process, although the involvement of glycoprotein Ib-IX remains somewhat debated. These observations reinforce the idea that familiar players in platelet hemostasis and thrombosis also contribute to inflammatory processes [48].

When platelet TLRs detect microbial species, platelet activation occurs, leading to degranulation and the release of various proinflammatory mediators. This release results in the discharge of approximately 300 proteins and biomolecules into the surrounding vasculature. CD154 (CD40 ligand), a potent secretory molecule, plays a key role in lymphocyte activation and has been extensively studied for its involvement in potentiating the adaptive immune response. Platelets are the primary source of soluble CD154 released upon platelet activation. In addition to CD154, platelets secrete antimicrobial proteins (thrombocidins), reactive oxygen species, and lytic enzymes, all of which help to enhance immune clearance.

Platelets also demonstrate the ability to recognize and release cytokines and chemokines in response to microbial threats, further boosting immune responses. They can engulf foreign particles, such as HIV particles and Staphylococcus aureus cells, into subcellular compartments. This internalization process occurs within the open canalicular system, and while not fully understood, it does not appear to result in microbial degradation or phagocytosis. Thus, platelets serve as storage units for a variety of bioactive molecules and enzymes while maintaining the capacity to consume invaders, suggesting that they may have evolved from an ancient granulocyte.

Beyond direct microbial defense, platelets assist primary immune cells in clearing pathogens. As seen in atherosclerosis, platelets adhere to activated or inflamed endothelium, where they facilitate leukocyte tethering to the vascular wall. This interaction enhances the migration of leukocytes into infected tissues, thereby aiding in infection clearance by increasing the number of white blood cells at the target site. Additionally, the interaction between platelets and Kupffer cells in the liver appears to be involved in the clearance of both bacterial cells and platelets during infection. Interestingly, platelets’ surface membrane glycoproteins, such as integrin αIIbβ3, glycoprotein Ib-IX, and FcγRIIa, are also implicated in microbial recognition. These receptors facilitate platelet activation and the release of secondary mediators, including platelet factor 4, which amplifies the activation process.

Neutrophils, which play a critical role in bacterial clearance, can release nuclear content into the bloodstream to capture pathogens. This results in the formation of neutrophil extracellular traps (NETs), which ensnare circulating microbes and limit their spread. Studies show that activated platelets, when adhering to neutrophils, can initiate NET formation, or NETosis. Interestingly, while lipopolysaccharides (LPS) can activate neutrophils, they are insufficient alone to trigger NET formation. However, when platelets are present, LPS can induce NETosis, although higher concentrations are required than those typically needed to activate neutrophils.

Although thrombus formation is often seen as a negative feature of platelet activity, recent research suggests that it may serve an important immune function. In response to microbial invasion, thrombogenesis is triggered alongside inflammation. The controlled formation of thrombi at select vascular sites creates a scaffolding that traps microbial particles, similar to the function of NETs. These “immunothrombi” are the result of multiple activating events converging on hemostatic players, offering a physiologically relevant response to infection [49].

Interestingly, the NETs produced with the help of platelets also facilitate coagulation. The negatively charged nucleobases in NETs can activate the contact pathway of coagulation, raising thrombin levels and promoting platelet activation. NETs also provide a platform for platelet docking and activation. Additionally, platelet engagement with microbes contributes to platelet stimulation and thrombosis. Immunothrombi not only trap migrating microbes but also serve as a base for tethering leukocytes, antimicrobial proteins, and lytic enzymes, localizing the immune response to clear the infection.

The formation of platelet–leukocyte interfaces enables platelets to further modulate immune responses. By releasing CD154, platelets enhance the adaptive immune response, and they can also help expand specific CD8+ T-cell clones by presenting antigens in the context of major histocompatibility complex I. Although there is still much to uncover about the role of platelets in immune cascades, the current body of evidence supports the view of platelets as an integral part of the immune system [50,51].

## 9. Clinical Treatment Strategies for Platelet Dysfunction

Antiplatelet agents are commonly used in the developed world. While these drugs provide significant benefits for patients with cardiovascular risk factors, they also necessitate protocols for managing unexpected or severe bleeding, particularly during surgery. The extent of platelet dysfunction is linked to bleeding risk, but discontinuing these agents can lead to rebound cardiovascular morbidity. Platelet dysfunction is also observed in other situations, particularly during cardiopulmonary bypass (CPB), where it has been associated with an increased risk of postoperative bleeding. In CPB, platelet dysfunction may result from the reduced availability of platelet agonists rather than intrinsic defects in the platelets themselves. CPB has also been shown to elevate platelet activation markers, such as thromboglobulin, platelet factor 4, and P-selectin.

When rapid reversal of antiplatelet therapy is needed, many guidelines recommend transfusing two to three adult doses of platelets. However, these recommendations are made with the understanding that there is limited evidence to fully support their effectiveness. Moreover, the optimal use of platelet transfusions has been questioned following the PATCH trial, which compared platelet transfusion to standard care in intracranial hemorrhage. This randomized trial found no benefit from platelet transfusion in reducing bleeding and even suggested that it might worsen outcomes, leading to increased mortality and poorer functional recovery. While the results of this trial may not be applicable to all situations involving platelet dysfunction, they highlight important concerns regarding the risk-to-benefit ratio of platelet transfusions.

Desmopressin (DDAVP), which has been used for nearly 40 years to treat congenital bleeding disorders, works by increasing plasma levels of von Willebrand factor, factor VIII, and intracellular platelet calcium/sodium ion concentrations. It also enhances the formation of procoagulant platelets and their adhesion to collagen under flow conditions. Desmopressin is recommended in several guidelines for managing bleeding in patients with platelet dysfunction or those taking antiplatelet agents. However, there has been no formal or systematic review assessing the sizes of the benefits and the risks associated with desmopressin use in this context. Potential side effects of desmopressin include hypotension, hyponatremia, facial flushing, and a theoretical risk of thrombotic events. The reviewed trials focused primarily on cardiac surgery, with six studies involving coronary artery bypass grafting (CABG), one involving aortic valve replacement, and three involving a combination of CABG and valve replacements. Platelet dysfunction was caused by antiplatelet agents in six trials, including aspirin and dual antiplatelet therapy, while four trials attributed dysfunction to cardiopulmonary bypass (CPB). Desmopressin was administered in various doses, with most trials using a 0.3 μg/kg intravenous dose, and it was given at different stages of surgery: preoperatively, intraoperatively (post-CPB), or postoperatively.

The trials included both prophylactic administration of desmopressin to prevent bleeding and its use in treating bleeding. Desmopressin was compared to placebo or standard care in most studies. The trials showed that desmopressin led to a reduced need for red blood cell transfusions compared to placebo, although the quality of evidence was deemed low. Regarding thrombotic events, no significant difference was found between desmopressin and placebo in terms of myocardial infarction, ischemic stroke, or venous thromboembolism, though the event rate was low overall [52].

One concern was an increased risk of clinically significant hypotension in patients receiving desmopressin, with a reported odds ratio of 9.78, though this risk was rare. The number needed to treat to cause one episode of hypotension was 17. Adverse events such as hyponatremia, seizures, chest tightness, and others were not commonly reported in the included trials. In addition to desmopressin, several other clinical treatment strategies are employed to manage platelet dysfunction, depending on the underlying cause and the clinical context. For patients experiencing platelet dysfunction due to antiplatelet therapy, platelet transfusions are commonly used to temporarily restore platelet function, especially in emergency or high-risk bleeding situations. However, the benefits of platelet transfusions, particularly in cases of antiplatelet therapy-related dysfunction, remain controversial, with some studies suggesting that the risk of thrombotic events may outweigh the benefits of transfusion.

In cases of congenital platelet disorders, such as Glanzmann thrombasthenia or Bernard–Soulier syndrome, platelet transfusions may be necessary to manage bleeding episodes, especially during surgeries or major trauma. For other specific disorders, such as uremic platelet dysfunction, dialysis may improve platelet function by removing uremic toxins from the bloodstream. Additionally, in patients with liver disease, which can cause platelet dysfunction due to thrombocytopenia and altered platelet production, interventions like platelet growth factors or thrombopoietin mimetics (e.g., eltrombopag) may be considered to stimulate platelet production and reduce the risk of bleeding.

Pharmacologic agents such as antifibrinolytics (e.g., tranexamic acid) are also used in conjunction with other treatments to stabilize clot formation and reduce excessive bleeding, particularly in surgeries where there is a known risk of significant blood loss. Tailoring treatment to the individual patient, considering the type and cause of platelet dysfunction, and monitoring for adverse effects are key components in optimizing clinical outcomes in patients with platelet-related bleeding or thrombotic risks.

NovoSeven (Eptacog Alfa [Activated]) is indicated for the treatment of bleeding episodes and for the prevention of bleeding associated with invasive procedures or surgical interventions in patients with Glanzmann’s thrombasthenia who have antibodies against GP IIb-IIIa and/or HLA and are currently or have previously been refractory to platelet transfusions [53].

***Glanzmann’s thrombasthenia treatment***

The recommended dose for treating bleeding episodes and preventing bleeding during surgical procedures or invasive interventions is 90 µg (range 80–120 µg)/kg at intervals of 2 h (1.5–2.5 h). To ensure effective hemostasis, at least three doses should be administered. The preferred route of administration is intravenous bolus injection, as continuous infusion may be less effective. For patients without contraindications, platelet transfusion remains the primary treatment option for Glanzmann’s thrombasthenia.

***von Willebrand disease (VWD) treatment***

For severe forms of von Willebrand disease (type 3) or patients who do not respond to desmopressin (DDAVP), treatment involves von Willebrand factor (vWF) and Factor VIII concentrates, such as Wilate and Immunate. Wilate contains human von Willebrand factor and human coagulation factor VIII. It is used for the prophylaxis and treatment of bleeding episodes or for surgical procedures in patients with von Willebrand disease when monotherapy with desmopressin (DDAVP) is ineffective or contraindicated. The ratio of vWF:RCo to FVIII:C is 1:1. In general, 1 IU/kg body weight of vWF:RCo and FVIII:C increases the plasma concentration by 1.5–2% of the normal activity level.

For effective hemostasis, the recommended dose is typically 20–50 IU Wilate/kg, which increases the plasma concentration of vWF:RCo and FVIII:C by approximately 30–100%. For type 3 von Willebrand disease, an initial dose of 50–80 IU Wilate/kg may be required, as maintaining appropriate plasma concentrations in these patients may necessitate higher doses compared to other types of VWD.

Immunate is a plasma-derived coagulation factor complex containing Factor VIII and von Willebrand factor. It is used for the treatment of bleeding episodes in von Willebrand disease patients with Factor VIII deficiency, particularly when no specific von Willebrand disease treatment is available or when desmopressin (DDAVP) monotherapy is ineffective or contraindicated [52,53].

## 10. Platelet Dysfunction in Neurological Diseases

Platelet dysfunction has been increasingly recognized as a potential contributor to the pathogenesis and progression of neurological diseases, including Alzheimer’s disease (AD) and Parkinson’s disease (PD). Both of these neurodegenerative disorders are associated with alterations in platelet function, which may have implications for disease progression, vascular health, and the management of related complications. In Alzheimer’s disease, platelet dysfunction is thought to be linked to amyloid-beta (Aβ) accumulation and tau pathology, which are hallmarks of the disease. Studies have shown that platelets in AD patients exhibit abnormal activation, aggregation, and impaired adhesion, which may contribute to the increased risk of cardiovascular events commonly observed in these patients. The altered platelet function could also be related to impaired microcirculation in the brain, promoting a state of vascular dysfunction that exacerbates cognitive decline. Additionally, the increased platelet aggregation observed in AD may contribute to thrombotic events, such as stroke, which are known to be more common in patients with AD [54].

In Parkinson’s disease, platelet dysfunction is also an area of growing interest. Platelets in PD patients show signs of altered function, with some studies indicating increased platelet aggregation and heightened activation in the presence of neuroinflammatory markers. This dysfunction may play a role in both the motor and non-motor symptoms of the disease, as well as in the increased risk of thromboembolic events, including stroke and deep vein thrombosis. The pathophysiology of platelet dysfunction in PD is complex and may be influenced by factors such as oxidative stress, neuroinflammation, and dopaminergic system degeneration. Additionally, the use of antiparkinsonian medications, such as dopamine agonists, may further alter platelet function, adding a layer of complexity to the management of bleeding or thrombotic risks in these patients. Platelet dysfunction in PD is thought to contribute to vascular changes in the brain that worsen neurodegeneration and may even affect the blood–brain barrier, facilitating neurodegenerative processes.

The relationship between platelet dysfunction and neurological diseases like Alzheimer’s and Parkinson’s is bidirectional, where platelet abnormalities can contribute to disease pathology, and conversely, neurodegenerative processes can influence platelet function. Understanding the role of platelets in these diseases is critical for developing new therapeutic strategies, especially as patients with these conditions often require careful management of both bleeding and thrombotic risks. Further research into platelet function in neurological diseases may provide insight into potential biomarkers for early diagnosis, prognosis, and the development of targeted therapies aimed at restoring normal platelet activity [55].

## 11. Conclusions

Platelet dysfunction plays a central role in both bleeding disorders and thrombosis, although its impact in each scenario is distinct.

In bleeding disorders, whether due to inherited conditions like Wiskott–Aldrich syndrome or Glanzmann thrombasthenia, or acquired conditions such as autoimmune platelet dysfunctions, the impairment of platelet function or reduced platelet numbers leads to inadequate clot formation, resulting in excessive or spontaneous bleeding. These dysfunctions may manifest in various forms, including abnormal platelet aggregation, defective platelet activation, or issues with platelet granules, leading to delayed or ineffective hemostasis.

In contrast, in thrombosis, platelet dysfunction can contribute to excessive platelet activation and aggregation, leading to the formation of abnormal thrombi (clots) within blood vessels. This can result in conditions such as deep vein thrombosis, pulmonary embolism, or myocardial infarction. In thrombosis, while platelets are typically hyperactive, their function still hinges on the balance between activation and inhibition. Conditions that disrupt this balance, such as certain genetic mutations or autoimmune responses, can increase the risk of inappropriate platelet aggregation, contributing to the pathogenesis of thrombotic events.

The early identification of platelet dysfunction and understanding of the underlying mechanisms are essential for effective treatment and management. The complex role of platelets in both hemostasis and thrombosis highlights the importance of maintaining platelet function within a narrow functional range, with both extremes leading to significant clinical complications.

## Figures and Tables

**Table 1:** The role of platelets in neoplasias, ET, and erythromelalgia.

Process/Condition	Role of Platelets	Mechanism	Implications
Tumorigenesis	Platelet-derived microparticles stimulate mitogen-activated protein kinases and MMP expression	Promotes tumor growth and invasion	Enhances tumor progression
Apoptosis Evasion	Platelets inhibit apoptosis via MMP-9 expression and mitochondrial uncoupling	Prevents cell death	Enhances tumor survival and progression
Chemoresistance	Platelets release growth factors and create a protective tumor microenvironment	Enhances resistance to chemotherapy	Reduces effectiveness of treatment
Angiogenesis	Platelets release VEGF, PDGF, and FGFs	Supports tumor vascularization	Promotes tumor growth and metastasis
Metastasis Facilitation	Platelets form a “cloak” around circulating tumor cells	Shields tumor cells from immune attack and shear stress	Enhances immune evasion and metastatic colonization
Cancer Stem Cell Support	Platelets promote EMT and cancer stem cell gene expression	Encourages self-renewal of cancer cells	Increases metastatic potential and worsens prognosis
Thrombosis and Bleeding in Essential Thrombocythemia (ET)	Elevated platelet count	Leads to arterial and venous thrombosis	Causes ischemia, infarction, spontaneous miscarriages, and bleeding
	Morphological platelet changes	Giant or hypogranular platelets reflect uncontrolled production in the bone marrow	Affects platelet function
	Platelet receptor alterations	Modifies adhesion and aggregation properties	Contribute to thrombosis and bleeding risk
	Impaired granule secretion (alpha and dense granules)	Affects the ability of platelets to support effective coagulation	Leads to bleeding tendencies
	Aggregability deficit	Reduces clot formation ability	Increases bleeding risk
	Platelet hyperreactivity	Increases aggregation and adhesion molecule expression (P-selectin)	Leads to thrombotic complications
Erythromelalgia	Platelet-mediated inflammation and arteriolar thrombosis	Excessive platelet activation in microcirculation	Causes vasodilation, inflammation, and microthrombosis
	Platelet hyperreactivity	Excessive release of serotonin, thromboxane A2, and platelet factor 4 (PF4)	Contributes to vasodilation, inflammation, and microthrombosis
	Formation of transient microthromboses	Hyperactive platelets cause repeated microthrombosis episodes, followed by compensatory reperfusion	Leads to vascular instability
	Interaction between hyperactive platelets and endothelial cells	Release of pro-inflammatory factors increases vascular permeability and triggers abnormal vasodilation	Results in severe burning pain and tissue congestion

**Table 2:** Gene mutations associated with Hermansky–Pudlak Syndrome (HPS).

HPS Subtype	Gene Mutated	Protein Complex Affected	Key Features	Pulmonary Fibrosis Risk
HPS-1	*HPS1*	BLOC-3	Severe bleeding, albinism, high fibrosis risk	High
HPS-2	*AP3B1*	AP-3	Immunodeficiency, neutropenia, early-onset fibrosis	High
HPS-3	*HPS3*	BLOC-2	Milder bleeding, albinism, no fibrosis	None
HPS-4	*HPS4*	BLOC-3	Similar to HPS-1, fibrosis risk	High
HPS-5	*HPS5*	BLOC-2	Milder bleeding, albinism, no fibrosis	None
HPS-6	*HPS6*	BLOC-2	Milder bleeding, albinism, no fibrosis	None
HPS-7	*HPS7*	BLOC-1	Rare, mild bleeding, albinism	Unknown
HPS-8	*HPS8*	BLOC-1	Rare, mild bleeding, albinism	Unknown
HPS-9	*HPS9*	BLOC-1	Rare, mild bleeding, albinism	Unknown
HPS-10	*HPS10*	BLOC-1	Rare, mild bleeding, albinism	Unknown

**Table 3:** Congenital platelet disorders and their mechanisms of dysfunction.

Congenital Platelet Disorders	Mechanism of Platelet Dysfunction
Glanzmann Thrombasthenia	Deficiency of glycoprotein IIb/IIIa → impaired platelet aggregation
Bernard–Soulier Syndrome	Deficiency of glycoprotein Ib/IX/V → impaired platelet adhesion
Von Willebrand Disease	Quantitative/qualitative deficiency of von Willebrand factor → impaired platelet adhesion
Paris-Trousseau Syndrome	Deletion of the long arm of chromosome 11 → abnormal megakaryocyte differentiation
Quebec Platelet Disease	Abnormal degradation of coagulation factor V due to overproduction of urokinase plasminogen activator (uPA) in platelets → hyperfibrinolysis
Hermansky–Pudlak Syndrome	Defect in dense granules → impaired ADP and serotonin release
Chediak–Higashi Syndrome	Defect in dense granules → impaired platelet secretion
Thrombocytopenia with Absent Radii (TAR) Syndrome	Deficiency of the RBM8A gene (affecting Y14 protein, essential for megakaryocyte function) → thrombocytopenia (<50,000/mm^3^), defective megakaryocyte maturation, impaired adhesion and aggregation, deficient release of pro-aggregant factors
Wiskott–Aldrich Syndrome	Mutations in the WAS gene, encoding the WASp protein essential for cytoskeleton organization in platelets → small platelets, thrombocytopenia (<50,000/mm^3^), impaired platelet aggregation
Congenital Afibrinogenemia	Absence of fibrinogen → impaired platelet aggregation
P2Y12 Receptor Deficiency	Deficiency of the P2Y12 receptor for ADP → impaired platelet aggregation
Collagen Receptor Deficiency (GPVI, α2β1)	Impaired platelet adhesion and activation
Alpha Granule Deficiency (Gray Platelet Syndrome)	Deficiency of alpha granules → impaired secretion of procoagulant factors
Cyclooxygenase-1 (COX-1) Deficiency	Defect in thromboxane A2 synthesis → impaired platelet aggregation

**Table 4:** Types of surgical interventions associated with platelet dysfunction.

Type of Surgery	Examples of Interventions	Mechanisms Involved in Platelet Dysfunction
Cardiovascular Surgery	Coronary bypass Cardiac valve replacement Heart transplant	Extracorporeal circulation Heparinization Systemic inflammation
Hepatic Surgery and Liver Transplant	Liver transplant Major liver resection	Liver failure Associated thrombocytopenia
Major Orthopedic Surgery	Hip or knee replacement Extensive spinal surgery Severe orthopedic trauma	Severe hemorrhage Inflammation Use of antifibrinolytic agents
Neurosurgery	Cerebral hemorrhages Cranial trauma requiring surgery Aneurysm treatment	Severe hemorrhage Hemodilution Thrombocytopenia
Major General Surgery	Extensive oncological surgery (large gastrointestinal resections) Pancreatectomy Esophagectomy	Severe hemorrhage Systemic inflammation DIC (Disseminated intravascular coagulation)
Thoracic Surgery	Pneumonectomy Pulmonary lobectomy Aortic dissection	Hypothermia Endothelial activation Use of heparin
Major Gynecological Surgery	Radical hysterectomy for cancer Surgical interventions for severe postpartum hemorrhage	Severe hemorrhage DIC Systemic inflammation
